# Biochar and bio-oil fuel properties from nickel nanoparticles assisted pyrolysis of cassava peel

**DOI:** 10.1016/j.heliyon.2022.e10114

**Published:** 2022-08-11

**Authors:** Titus Chinedu Egbosiuba

**Affiliations:** Chemical Engineering Department, Chukwuemeka Odumegwu Ojukwu University, Uli Campus, Anambra State, Nigeria

**Keywords:** Biomass, Biochar, Bio-oil, Biogas, Dried cassava peels, Slow pyrolysis, Nickel nanoparticles

## Abstract

Direct biomass usage as a renewable fuel source and substitute for fossil fuels is discouraging due to high moisture, low energy density and low bulk density. Herein, thermogravimetric analysis (TGA) was conducted at various heating rates to determine peak decomposition temperatures for the dried cassava peels (DCP). The influence of pyrolysis temperature (300, 400, 500 and 600 °C) and heating rates (10, 20 and 30 °C/min) on the nickel nanoparticles catalyzed decomposition of DCP to produce biochar, bio-oil and biogas was investigated and characterized. The results revealed higher biochar (CBC) yield of 68.59 wt%, 62.55 wt% and 56.92 wt% at lower pyrolysis temperature of 300 °C for the different heating rates of 10, 20 and 30 °C/min. The higher carbon content of 52.39, 53.30 and 55.44 wt% was obtained at elevated temperature of 600 °C and heating rates of 10, 20 and 30 °C/min, respectively. At the pyrolysis temperature of 600 °C and heating rates of 10, 20 and 30 °C/min, the optimum yield of bio-oil (24.35, 17.69 and 18.16 wt%) and biogas (31.35, 42.03 and 46.12 wt%) were attained. A high heating value (HHV) of 28.70 MJ/kg was obtained for the biochar at 600 °C. Through the TGA, FTIR and HRSEM results, the thermal stability, hydrophobicity and structural changes of DCP and CBC samples were established. Similarly, the thermal stability of CBC samples increased with increasing pyrolysis temperature. Biochar with optimum fuel properties was produced at 600 °C due to the highest carbon content and high heating value (HHV). Improved kinematic viscosity (3.87 mm^2^/s) and density (0.850 g/cm^3^) were reported at the temperature of 300 °C and heating rate of 30 °C/min, while a higher pH (4.96), HHV (42.68 MJ/kg) and flash point (53.85 min) were presented by the bio-oil at the temperature of 600 °C and heating rate of 30 °C/min. Hence, DCP produced value-added biochar and bio-oil as renewable energy.

## Highlights

• Nickel nanoparticles successfully catalyzed the pyrolysis of CP biomass.
• Temperature and heating rates affected the yield of pyrolysis products.
• Fixed carbon content increased rapidly with temperature increase.
• The HHV of both biochar and bio-oil was higher than the DCP biomass.
• The fuel properties of biochar and bio-oil improved rapidly through NiNPs catalyzed pyrolysis.

## Graphical abstract

**Figure:**
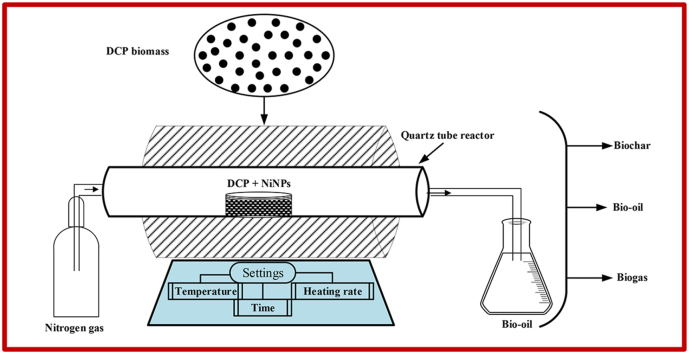

## 1 Introduction

Over the years, significant growth in the consumption of fossil fuels like oil, gas and coal has been recorded due to rapid demographic development, awareness of environmental responsibilities and global modernization, thereby increasing the universal demand for energy (R. K. Singh et al., 2021). Although fossil fuels represent a suitable energy source but the overt dependence on their use for power generation puts pressure on the finite fossil fuel reserve depletion, enhances global change in the climate, air pollution concerns and contributes immensely to global warming (Sriram and Swaminathan, 2018). In recent times, a study conducted by International Energy Association revealed that by 2040, half of the crude oil reserve around the globe would have been depleted (Sriram and Swaminathan, 2018). Hence, avid demand for fossil fuels has resulted in environmental challenges such as acid rain, biodiversity loss, soil pollution, global warming due to harmful greenhouse gas emissions from the combustion of fossil fuels and water pollution due to oil spillage (Pant and Rai, 2021). The associated problems of fossil fuel usage have led to increased research, development and utilization of sustainable alternative fuels that are renewable and environmentally friendly (Karuppasamy Vikraman et al., 2021; Rasam et al., 2022; S. Wang et al., 2022).

Remarkably, other sources of renewable energy for instance solar, wind and hydropower have been used to generate heat and power, but biomass has been regarded as the most dominant renewable resource that not only generates heat and power but equally produces renewable liquid fuels, solid fuels, gaseous fuels and biobased chemicals for various industries (Hu et al., 2021; Salvilla et al., 2020; Singh et al., 2020b). The overall increase in global population, demand for food and agricultural activities have enhanced the rating of biofuels from agricultural biomass wastes as a viable renewable energy resource. Sequel to that, researchers have extensively explored biomass and biowastes for the production of energy, potential carbon neutral fuels and bio-based chemicals (Angın, 2013; Singh et al., 2020b). Biomass is described as organic matter obtained from living organisms that are rich in cellulose and organic compounds used as a fuel source and production of chemical products (Basu, 2018a; Kaur et al., 2018). For the generation of power, biomass can be directly used as a replacement or co-combusted with fossil fuel, thereby reducing the overall carbon emissions since the emitted carbon during the energy conversion process is normalized by the uptake of carbon during the growth of the plant (Selvarajoo et al., 2022). In reality, raw biomass is not attractive as a fuel because of its high moisture content, small energy density and low bulk density in comparison to fossil fuels. It is important to note that the overall combustion efficiency is greatly reduced by the high moisture content present in the fuel. Then again, low bulk density results in a higher cost of transportation per unit of energy (Chong et al., 2019; Mlonka-Mędrala et al., 2021). Therefore, it is paramount to convert the raw biomass using thermal methods to products with improved fuel properties.

Among the thermochemical processes for the conversion of biomass such as pyrolysis, gasification, combustion and liquefaction, pyrolysis is predominantly preferred due to low energy requirements, high energy recovery and production of an extensive spectrum of products (Angin et al., 2013; Basu, 2018b,c; Basu et al., 2018; Joshi and Seay, 2020; Patra et al., 2021; Singh et al., 2020b). Pyrolysis is the thermal decomposition of biomass by the application of heat in the absence of oxygen to produce solid, liquid and gaseous products, particularly in the pyrolysis temperature range of 300–700 °C (Al-Rumaihi et al., 2021; Basu, 2018b; Uzun and Yaman, 2017). As obtained from the biomass pyrolysis, the solid and liquid products are known as biochar and bio-oil, while the gaseous products are regarded as biogas or syngas containing carbon dioxide (CO_2_), carbon monoxide (CO), methane (CH_4_) and hydrogen (H_2_) (Saravanan et al., 2021; Selvarajoo et al., 2022). The fuel properties of the products are highly dependent on the pyrolysis conditions like temperature, heating rate, residence time and biomass particle size. Pyrolysis is classified into fast and slow pyrolysis. In fast pyrolysis, higher bio-oil and biogas yields are obtained at high heating rates and low residence time to hinder unstable volatile material's secondary reactions (Angın, 2013). On the other hand, slow pyrolysis produces higher biochar yield at a low heating rate and high residence time to enhance secondary char formation (Patra et al., 2021; Soka and Oyekola, 2020a). Notably, liquid and gaseous products have limitations in their applications. The direct usage of bio-oil as fuel is due to its high instability and complexity, while biogas requires further separation and purification with complex treatment methods (Selvarajoo et al., 2022). Specifically, biochar emanating from biomass has broad areas of applications (Shrivastava and Srivastava, 2021).

Furthermore, biochar is a highly porous and carbon-rich solid used predominantly as fuel which had been widely investigated as a result of its vast applications in different fields (Howell et al., 2021; Muhammad et al., 2022; Peng et al., 2021; Sato et al., 2020; Singh et al., 2021). Although biochar can be utilized as an adsorbent for wastewater treatment (Della et al., 2020), it is also an effective material in carbon sequestration and soil enrichment to improve the nutrients and water holding capacity of the soil (Selvarajoo et al., 2022). Overall, biochar possesses great potential as a renewable fuel alternative in energy production due to its high carbon and energy composition (Chong et al., 2019; Postawa et al., 2022). Some researchers have reported that good fuel properties of biochar with an enhanced combustion characteristic and performance in comparison to raw biomass (Basu, 2018a; Kaur et al., 2018; Mishra and Mohanty, 2018; Selvarajoo et al., 2022; Singh et al., 2020b). Additionally, biochar properties differ from that of activated carbon but biochar is undeniably used effectively to prepare activated carbon of appropriate surface area and pore structures (Angin et al., 2013). On the other hand, the high viscosity index and ash content with a low calorific value, acidity and instability have posed great limitations to the application of bio-oil. Improved understanding of bio-oil production will assist researchers to reduce the challenges associated with bio-oil and optimize process parameters that will enhance its application as a substitute for diesel fuels (Li et al., 2021).

Generally, the production of biochar and bio-oil can be conducted from different waste biomass sources such as almond shell, corn cob, rice husk, sugarcane bagasse, groundnut shell, poplar sawdust, food waste, Crofton weed, citrus peel, coffee husk, palm kernel shell, walnut shell and cassava rhizome (Cheng et al., 2021, 2022; Egbosiuba et al., 2020a; Ifa et al., 2020; Islam et al., 2021; Kalair et al., 2021; Morales et al., 2021; Nkomo et al., 2021; Promraksa and Rakmak, 2020; Quillope et al., 2021; Rueangsan et al., 2021; Saleem, 2022; Selvarajoo et al., 2022; Soka and Oyekola, 2020b). The quality of the biochar can be determined by physicochemical parameters such as volatile matter, percentage carbon content, fixed carbon, ash content and higher heating value (HHV). Although Zhang et al. (2022a) and Duan et al. (2022) have investigated the activities of metallic zinc, iron, nickel, zeolite and activated carbon on the pyrolysis of lignocellulosic, plastic and corncob waste, no study has been found to study NiNPs catalyzed pyrolysis of cassava peel for different product distributions.

Cassava (*Manihot esculenta crantz*) is a perennial woody shrub that consists of edible roots and is a fast-expanding staple food crop (FAOSTAT, 2021; Ikuemonisan et al., 2020). At the moment, the global cassava production capacity stood at about 278 million MT and Africa accounts for about 192 million MT (64%) (SAHEL, 2021). In all, Nigeria is the largest producer of cassava in the world with about 60 million MT (19.4% of global production) (FAOSTAT, 2021). The majority of cassava produced in Nigeria is consumed locally as traditional foods while others are utilized in the industries to produce products like ethanol, starch, flour, animal feed and sorbitol (SAHEL, 2021). As such, the processing of the cassava roots creates about 15 million MT of cassava peels per year, thereby leading to environmental challenges and health implications (Adeboye et al., 2021; Iheanacho, 2021). Cassava peels are lignocellulosic agricultural waste consisting primarily of fibres and essential oils. The availability of cassava peel is a great asset over some biomass materials that are not readily available for biochar and bio-oil production. Therefore, it is essential to recover energy, fibres and oils that can be used in different industries such as agricultural, energy, water, cosmetics, pharmaceuticals and confectioneries industries (Kaur et al., 2018; Selvarajoo et al., 2022). For this purpose, cassava peel is very suitable for thermochemical conversion to various products.

The novelty of this study lies in obtaining the thermogravimetric assisted degradation temperature of cassava peel. This research also investigated the effectiveness of NiNPs catalysts in the promotion of catalytic pyrolysis of DCP to achieve higher quality biochar and bio-oil. The use of NiNPs as a catalyst has not yet been investigated in the pyrolysis of DCP. Therefore, this study provides profound knowledge on the potential synergistic influence of NiNPs for improved biochar and bio-oil quality through accelerated heating. In addition, the product yields of cassava peel pyrolysis, proximate analysis, elemental analysis, high heating value (HHV), fuel ratio and fuel properties were also evaluated at different heating rates and temperatures. Overall, this study complies with the principles of sustainable development and circular economy by the catalytic conversion of the waste biomass into biochar and bio-oil as an alternative fuel source, waste management improvement and environmental protection.

## 2 Materials and methods

Cassava peels (CP) were procured from cassava processing plant, Neni Anambra State Nigeria. The collected CP were washed with distilled water before drying under the sun for a week for the reduction of the moisture content. The dried CP (DCP) was cut and screened to have an average particle size of 2 mm for the pyrolysis experiments because of the suitability of smaller particle sizes to ensure superior heat transfer efficiency. Then, the DCP samples were kept safe in a glass container to prevent the absorption of moisture. The measurements of the sample weight were conducted after cooling to 20–40 °C in a desiccator. The NiNPs catalysts used in this study were produced in our previous study reported elsewhere (Egbosiuba et al., 2022).

### 2.1 Thermogravimetric analysis of DCP

A thermogravimetric analyzer (TGA, PerkinElmer, UK) was employed to investigate the thermal degradation behaviour of DCP (Oboh et al., 2018). In detail, 10 mg of CP sample was placed into the crucible of the thermogravimetric analyzer and performed at temperatures of 30–900 °C and heating rates of 10, 20 and 30 °C/min under a nitrogen flow rate of 50 mL/min as a non-reacting inert gas. The experiments were repeated in triplicates to ensure accurate data reproducibility. The data obtained from the TGA experiment were instrumental in the determination of the pyrolysis temperature range of the CP sample.

### 2.2 Pyrolysis experiments of DCP

**Figure 1:**
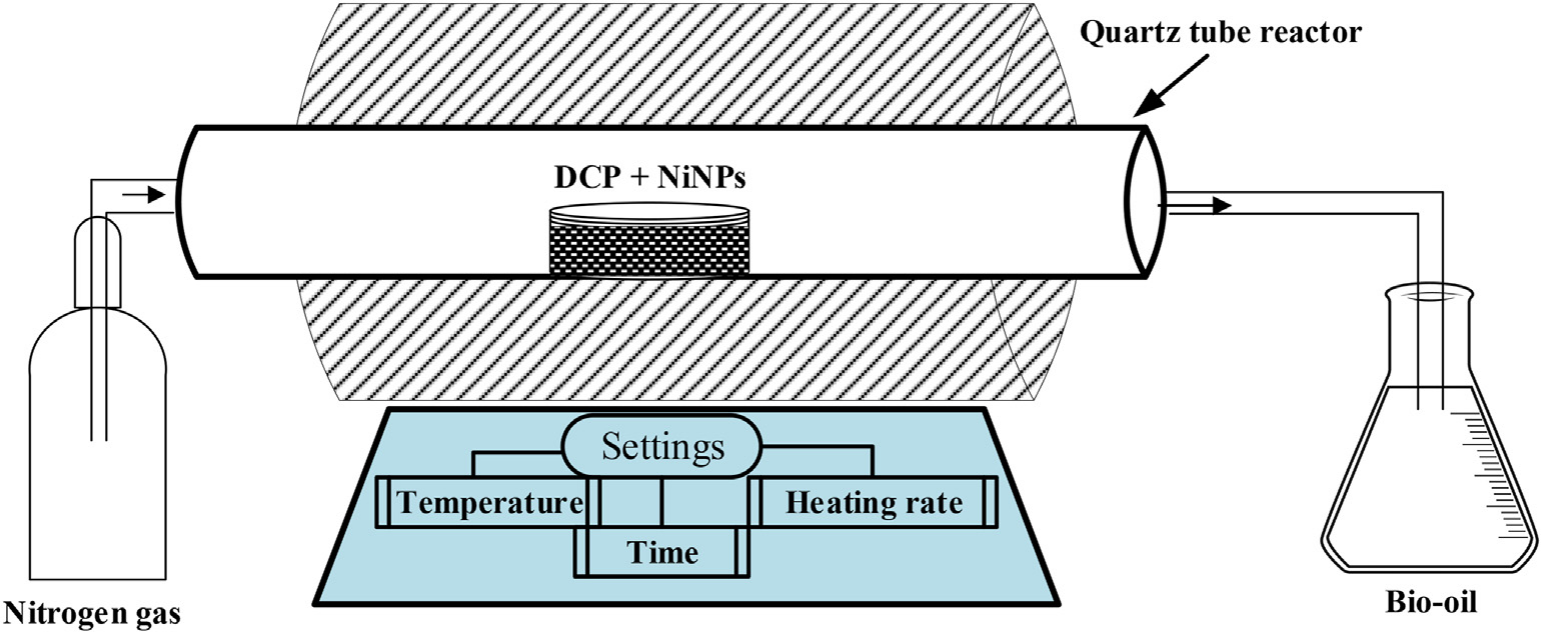
Pyrolysis experimental setup schematic representation.

The pyrolysis experiments were conducted in the tubular furnace using the methods reported elsewhere with a little modification (Li et al., 2022; Selvarajoo et al., 2022; Yang et al., 2021). Briefly, pyrolysis tests were performed by measuring 50 g of DCP and 5 mg of NiNPs samples in a crucible and placed inside the quartz tube reactor of the tubular furnace (Carbolite, CTF12/100/900) as shown in Figure 1.

The experiment was carried out at the temperatures of 300, 400, 500 and 600 °C at the heating rate of 10, 20 and 30 °C/min under a steady nitrogen flow rate of 50 mL/min to maintain an inert environment for 6 h. At the end of the process, the heating of the reactor was stopped and cooling commenced until ambient temperature. The experiments were repeated three times to ensure the accuracy of the data repeatability. The pyrolyzed biochar was withdrawn from the furnace and weighed to evaluate the biochar yield using Eq. (1) (Selvarajoo et al., 2022) and the obtained biochar samples at different temperatures labelled CBC-300 °C, CBC-400 °C, CBC-500 °C and CBC-600 °C. Similarly, the bio-oil was collected in a filtering flask placed at the opposite end of the tubular furnace and weighed to determine the bio-oil yield using the mathematical relationship in Eq. (2) (Peng et al., 2021). On the other hand, the biogas yield was evaluated as the difference between biochar and bio-oil yield using Eq. (3) (Li et al., 2022).

(1) $$\text{Yield ​of ​Biochar}\;\left(\%\right)=\frac{\text{Weight ​of ​solid ​product}}{\text{Weight ​of ​sample}}\times 100\%$$

(2) $$\text{Yield ​of ​Bio}-\text{oil}\;\left(\%\right)=\frac{\text{Weight ​of ​liquid ​product}}{\text{Weight ​of ​sample}}\times 100\%$$

(3) YieldofBiogas(%)=100%−YieldofBiochar(%)−YieldofBio-oil(%)

### 2.3 Characterization of biochar and bio-oil

#### 2.3.1 Proximate and elemental analysis

In this study, the proximate analysis and elemental analysis were determined to obtain information on the possibility of its conversion to value-added products. As such, the proximate analysis such as moisture content (MC), volatile matter (VM) and ash content (AC) were evaluated by the American Society for Testing and Materials (ASTM) standards like D3173, D3174 and D3175, respectively.

##### 2.3.1.1 Moisture content analysis

Moisture content (MC) is defined as the ratio of moisture to the weight of the solid fuel on a dry basis (Wang et al., 2022). Briefly, the MC was determined using ASTM D-3173 by oven drying 10 g of the samples placed in a crucible without a lid at the temperature of 105 °C or 110 °C for 1 h. The MC (%) was determined using Eq. (4) (Ifa et al., 2020).

(4) $$\text{MC}\;\left(\%\right)=\frac{{\text{w}}_{0}-{\text{w}}_{1}}{\text{w}}\times 100\%$$

##### 2.3.1.2 Volatile matter analysis

To determine the volatile matter (VM) using ASTM D-3175, 10 g of the sample was measured into a crucible covered with the lid and placed in a furnace before heating to 700 °C for 0.5 h (Afessa et al., 2022). The lost weight (A) due to volatile matter decomposition and volatile matter content was examined using Eqs. (5) and (6), respectively (Ifa et al., 2020).

(5) $$\text{Lost}\;\text{weight}\;\left(\%\right)=\frac{{\text{w}}_{0}-{\text{w}}_{1}}{\text{w}}\times 100\%$$

(6) VM(%)=lostweight−moisturecontent

##### 2.3.1.3 Ash content analysis

To investigate the ash content (AC) defined as the obtained residual mass after heating a solid fuel to a constant weight. It was evaluated using ASTM D-3174 standard by measuring 10 g of the sample into a crucible without a lid and placed into a furnace before heating at 900 °C for 7 min (Afessa et al., 2022). In the end, the ash content was calculated using Eq. (7) (Ifa et al., 2020).

(7) $$\text{AC}\;\left(\%\right)=100-\frac{{\text{w}}_{0}-{\text{w}}_{1}}{\text{w}}\times 100\%$$

in all, W_0_ (g) and W_1_ (g) represent the weight of the sample and crucible before and after drying, while W (g) is the initial weight of the sample.

##### 2.3.1.4 Fixed carbon content determination

To obtain the fixed carbon (FC), the sum of other proximate parameters (MC, VM and AC) were deducted from 100 as shown in Eq. (8) (Malucelli et al., 2019; Yang et al., 2021)

(8) FC(wt%)=100%−(MC+VM+AC)

##### 2.3.1.5 Elemental analysis

The ultimate analysis was conducted to establish the contents of carbon (C), hydrogen (H), nitrogen (N) and oxygen (O) present in the DCP and biochar using an elemental analyzer (Vario MC, Elementary, Germany). While C, H and N contents were determined from the elemental analyzer, O content was calculated using Eq. (9) (Sakhiya et al., 2021)

(9) O%=100%−(C%+H%+N%)

#### 2.3.2 Determination of fuel characteristics of biochar

Specifically in this study, the higher heating value (HHV) of the dried cassava peels (DCP) and biochar were evaluated using a bomb calorimeter (Parr Instruments, 6100). In this procedure, 1 g each of the ground powdered samples of DCP and biochar were ignited in an oxygen environment in the enclosure of the bomb cylinder. The samples were allowed to undergo complete combustion and the total heat released was recorded as the HHV.

In addition, the fuel ratio, energy yield and thermal stability of the DCP and biochar were evaluated using Eqs. (10), (11), and (12) (Baghel et al., 2022; Malucelli et al., 2019; Selvarajoo et al., 2022), respectively.

(10) $$\text{Fuel}\;\text{Ratio}=\frac{\text{FC}}{\text{VM}}$$

(11) $$\text{Energy}\;\text{yield}\;\left(\%\right)=\frac{\text{weight}\;\text{of}\;\text{biochar}\times \text{HHV}\;\text{of}\;\text{biochar}}{\text{weight}\;\text{of}\;\text{dried}\;\text{biomass}\times \text{HHV}\;\text{of}\;\text{dried}\;\text{biomass}}\times 100\%$$

(12) $$\text{Thermal}\;\text{stability}=\frac{\text{FC}}{\text{FC}+\text{VM}}$$

#### 2.3.3 Functional group analysis

Fourier transform infrared (FTIR) spectrometer (PerkinElmer, FTIR Frontier, UK) was used to conduct the chemical functional groups present in the DCP and the obtained biochar (CBC). The recording of the spectra was carried out in a wavelength range of 400–4000 cm^−1^ using the transmittance technique.

#### 2.3.4 Surface morphology analysis

The surface morphology of the DCP and CBC (at different temperatures) was evaluated using high-resolution scanning electron microscopy (HRSEM). The images from the analysis were captured at different magnifications in the range of 100x to 5000x in scanning mode.

#### 2.3.5 Determination of fuel characteristics of bio-oil

The obtained bio-oil was analyzed by ASTM standards (ASTM D975, 2013). The bio-oil used for the characterization was obtained after cooling by the condenser. Prior to the analysis, the bio-oil was centrifuged at 3000 rpm for 10 min. Generally, the physical and chemical properties of bio-oil include viscosity, specific gravity, pH, flash point, density, solubility, water content, residual carbon content, volatilization, solubility, thermal conductivity, specific heat capacity and HHV. However, viscosity, specific gravity, pH, flash point and HHV were only determined in this study using the standard procedures reported elsewhere (Li et al., 2021; Rueangsan et al., 2021; Tesfa et al., 2013).

## 3 Results and discussion

### 3.1 Thermogravimetric analysis

**Figure 2:**
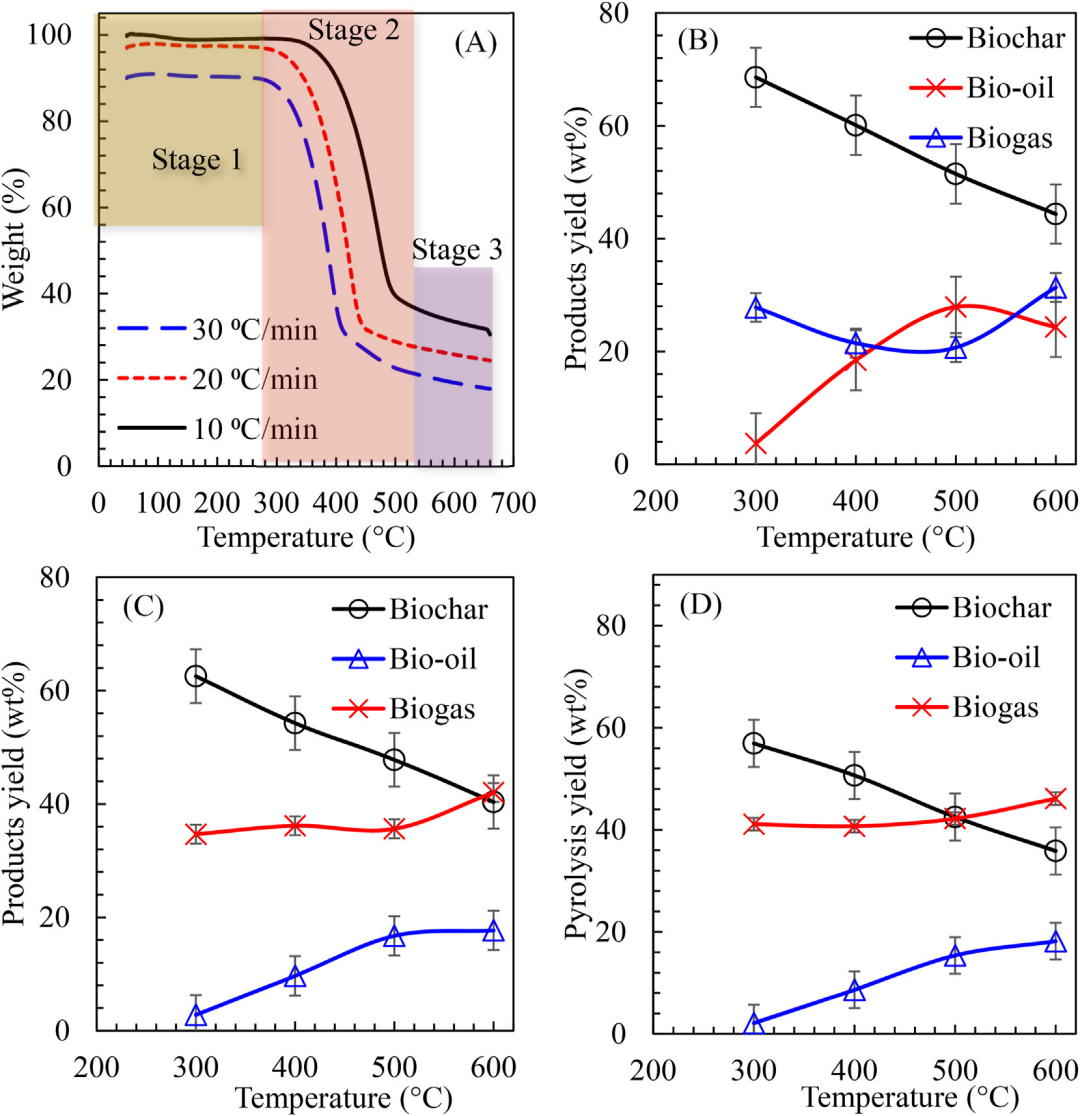
(A) Thermogravimetric curves of DCP for the heating rates of 10, 20 and 30 °C/min; Influence of temperature on the pyrolysis products at (B) 10 °C/min; (C) 20 °C/min and (D) 30 °C/min.

The thermogravimetric (TG) curves of the DCP obtained at various heating rates are shown in Figure 2(A). From the TG profile, a similar pyrolysis degradation trend was observed for the different heating rates and three stages of DCP degradation were observed between the temperature ranges of 50–350 °C, 350–500 °C and 500–600 °C. As can be seen from 50 to 350 °C, the weight loss of the DCP was evaluated as 1.35, 4.93 and 2.41% for the heating rates of 10, 20 and 30 °C/min. The observed weight loss at this stage may be ascribed to loss of moisture and release of light compounds from DCP due to the reduced mass loss (Hu et al., 2021; Singh et al., 2020a).

The mass loss at this stage is small, close to the moisture content and the decomposed mass was not converted to value-added products (Singh et al., 2021). Further thermal decomposition of DCP was revealed in the temperature range of 350–500 °C which corresponds to the weight loss of 59.49, 66.25 and 67.02% at the peak temperatures of 500, 480 and 450 °C for the heating rates of 10, 20 and 30 °C/min, respectively. The observed maximum weight loss at this stage could be linked to the removal of volatile matter (such as hydrocarbons, H_2_, CO, CH_4_ and incombustible gases (CO_2_, N_2_)) particularly lignocellulosic materials such as cellulose, hemicellulose and lignin components (Al-Rumaihi et al., 2021; Liu et al., 2020). The decomposed volatile matter in this region is mostly converted to value-added products such as bio-oil and gaseous mixture (Hu et al., 2021). Notably, hemicellulose thermal stability is lower compared to cellulose and would decompose into lighter compounds before cellulose, thus both decompose at temperatures below 450 °C (Selvarajoo et al., 2022). However, lignin possesses better thermal stability due to its complex structure, thereby decomposing over a wide temperature range of 200–900 °C (Selvarajoo et al., 2022).

In the third decomposition stage above 500 °C, the formation of biochar was noticeable and mass decomposition was evaluated as 5.87, 3.0 and 3.36% for the heating rates of 10, 20 and 30 °C/min, respectively. The observed TG curve was almost horizontal due to the degradation of lignin, minerals and produced char after devolatilization (Al-Rumaihi et al., 2021; Hu et al., 2021). At the end of the pyrolysis experiments, the remaining mass of the final residue for the heating rates of 10, 20 and 30 °C/min was obtained as 33.55, 25.92 and 19.40%, respectively. From the TGA results, it is evident that the majority of the organic materials in DCP were decomposed before reaching 500 °C and weight loss above this temperature was relatively stable. Consequently, pyrolysis temperatures of 500 ± 200 °C were suggested for effective decomposition of cassava peel biomass for high-quality biochar with attractive properties.

### 3.2 Effect of temperature and heating rate on the bio-products yield

The obtained yields for the biochar, bio-oil and biogas from the pyrolysis of DCP as a function of temperature and heating rates are shown in Figure 2(B-D). Accordingly, the effects of temperature and heating rates on the pyrolysis product distribution were very significant. As shown in Figure 2(B), increasing the pyrolysis temperature from 300 to 600 °C resulted to decrease in the biochar yield from 68.59 to 44.36 wt%, 62.55 to 40.34 wt% and 56.92 to 35.85 wt% for the heating rates of 10, 20 and 30 °C/min, respectively. The observed decrease in the yield of biochar as the temperature increased corroborated with the literature and may be attributed to the increased removal of volatiles during rapid lignocellulosic materials decomposition and also due secondary decomposition of primary char residues (Mohd Hasan et al., 2019; Selvarajoo et al., 2022). Hence, the higher biochar yield at a lower temperature may be due to partial pyrolysis or incomplete decomposition of the DCP material (Angin et al., 2013). Correspondingly, it can be inferred that a lower pyrolysis temperature is preferable to obtain a higher biochar yield, but the temperature should be high enough to enhance the complete pyrolysis of biomass into biochar. Similarly, heating rates were significant at lower temperatures, while a reduction trend similar to biomass yields was noticeable at higher temperatures.

Typically shown in Figure 2(C), the yield of bio-oil increased from 3.72 to 24.35 wt%, 2.80 to 17.69 wt% and 2.11 to 18.16 wt% for the heating rates of 10, 20 and 30 °C/min as the temperature was increased from 300 to 600 °C. The trend of this result showed agreement with previous studies that reported an increase in the yield of bio-oil as the pyrolysis temperature increased up to the temperature of 400–650 °C, where a further increase in pyrolysis temperature corresponds to a decrease in bio-oil yield (Selvarajoo and Oochit, 2020). Above all, the yield of biogas increases with pyrolysis temperature due to the occurrence of secondary cracking reactions, thereby leading to higher forms of gas products and lower molecular weight compounds (Li et al., 2022). Particularly in this study, the yield of biogas at the temperature of 300–600 °C is presented in Figure 2(D) increased from 27.81 to 31.35 wt%, 34.67 to 42.03 wt% and 41.10 to 46.12 wt% for the heating rates of 10, 20 and 30 °C/min. Noticeably, the heating rates had a negligible influence on the product distribution. The enhanced yield of biogas compared to bio-oil at higher pyrolysis temperatures was very significant and corresponds to the literature (Li et al., 2022; Selvarajoo and Oochit, 2020).

The preparation reaction of NiNPs (zero-valent nickel) and its interaction with DCP in the catalyzed pyrolysis process are indicated in Eqs. (13), (14), and (15) (Zhang et al., 2022b). Initially, the amorphous Ni (Ni(OH)_4_) was formed at a low temperature as shown in Eq. (13). In the calcination process of Ni(OH)_4_ at 500 °C, the amorphous Ni transformed to a zero-valent NiO (NiNPs) as presented in Eq. (14). Further interaction of the NiNPs with the DCP in the pyrolysis tube reactor at elevated temperature (600 °C) enhanced the production of highly carbonaceous biochar and bio-oil of improved fuel properties due to the reduction of the catalyst to metallic Ni by carbon, CO and H_2_ as expressed in Eq. (15) (Wang et al., 2022; Zhang et al., 2022b).

(13) $${\text{NiSO}}_{4}+2{\text{H}}_{2}\text{O}\to {\text{Ni}\left(\text{OH}\right)}_{4}+{\text{H}}_{2}\text{S}\;\text{T}\leq 110\;{}^{\circ}\text{C}$$

(14) $${\text{Ni}\left(\text{OH}\right)}_{4}\to {\text{NiO/Ni}}_{2}{\text{O}}_{3}\;\text{T}\leq 500\;{}^{\circ}\text{C}$$

(15) $$\text{NiO}/{\text{Ni}}_{2}{\text{O}}_{3}+\left(\text{C},\text{CO},{\text{H}}_{2}\right)\to \text{Metallic}\;\text{Ni}\;\text{T}\geq 600\;{}^{\circ}\text{C}$$

The observed nature of the DCP and NiNPs interaction indicates that charring reactions and formation of micropores occurred during the catalyzed pyrolysis to obtain CBC at various temperatures and heating rates. Above all, biochar and bio-oil were successfully produced using NiNPs as a catalyst in the pyrolysis technique.

Overall, the mixture of DCP with NiNPs significantly decreased the yield of biochar and increased the yield of bio-oil and gas respectively. Hence, NiNPs catalyzed pyrolysis of DCP favoured oil and gas production showed a similar trend in a conventional pyrolysis process, whereby hydrogen and gas yield is enhanced with the resultant reduction in biochar yield (Ghodake et al., 2021; Mohamed et al., 2021). The decline in the yield of biochar may be attributed to a higher average heating rate of the catalyzed DCP which stimulated deoxygenation and cracking reactions, thus producing more gaseous compounds than the solid and liquid compounds as presented in Eqs. (16), (17), (18), (19), and (20). The NiNPs catalyst also enhanced the dry reforming reaction of methane cracking as shown in Eqs. (16) and (17) (Mohamed et al., 2021, 2022).

(16) $${\text{CH}}_{4}\;\left(\text{g}\right)+{\text{H}}_{2}\text{O}\;\left(\text{g}\right)↔\text{CO}\;\left(\text{g}\right)+3{\text{H}}_{2}\;\left(\text{g}\right),\;\Delta {\text{H}}_{298\;\text{K}}=206\;\text{kJ/mol}$$

(17) $${\text{CH}}_{4}\;\left(\text{g}\right)+{\text{CO}}_{2}\;\left(\text{g}\right)↔2\text{CO}\;\left(\text{g}\right)+2{\text{H}}_{2}\;\left(\text{g}\right),\;\Delta {\text{H}}_{298\;\text{K}}=260.5\;\text{kJ/mol}$$

(18) $$\text{C}\;\left(\text{s}\right)+{\text{CO}}_{2}\;\left(\text{g}\right)↔2\text{CO}\;\left(\text{g}\right),\;\Delta {\text{H}}_{298\;\text{K}}=173\;\text{kJ/mol}$$

(19) $$\text{C}\;\left(\text{s}\right)+{\text{H}}_{2}\text{O}\;\left(\text{g}\right)↔\text{CO}\;\left(\text{g}\right)+{\text{H}}_{2}\;\left(\text{g}\right),\;\Delta {\text{H}}_{298\;\text{K}}=132\;\text{kJ/mol}$$

(20) $$\text{Tar}\;\left(\text{g}\right)+{\text{CH}}_{4}\;\left(\text{g}\right)+{\text{C}}_{\text{m}}{\text{H}}_{\text{n}}\;\left(\text{g}\right)+{\text{H}}_{2}\text{O}\;\left(\text{g}\right)+{\text{H}}_{2}\left(\text{g}\right)$$

### 3.3 Proximate and elemental analysis of biochar

Remarkably, it was obvious that the dried cassava peel (DCP) and biochar (CBC samples) were predominantly composed of volatile matter and fixed carbon, whereby the biomass had a higher volatile matter composition compared to the biochar, while the biochar had a higher fixed carbon content than the biomass. Generally, combusting biomass does not always translate to a high energy output because of high contents of moisture and volatile matter as shown in Table 1.

**Table 1:** Physical and chemical properties of dried cassava peel (DCP) biomass.

Characterization	Parameters	Values
Proximate characteristics (wt%)	Moisture content	10.32
	Volatile matter	71.66
	Ash content	0.25
	Fixed carbon	14.77
Ultimate characteristics (wt%)	Carbon (C)	42.01
	Oxygen (O)	38.60
	Hydrogen (H)	5.21
	Nitrogen (N)	1.05
	Sulfur (S)	0.20
	H/C	0.12
	O/C	0.92
High Heating Value (HHV, MJ/kg)		18.30

Fuels with high contents of volatile matter require a larger quantity of secondary air to achieve combustion effectiveness. Therefore, additional thermal treatment of the DCP is required before usage for energy conversion. In this study, a reduction in the volatile matter content from 74.66 to 60.60, 57.80 and 55.60 wt% for the heating rates of 10, 20 and 30 °C/min was observed after pyrolysis of DCP to biochar at 300 °C as shown in Table 2. Further increase in temperature up to 600 °C decreased the volatile matter content to 41.82, 35.10 and 20.90 wt% for the heating rates of 10, 20 and 30 °C/min. The revealed decreasing trend in the contents of volatile matter may be ascribed to the progressive volatiles removal due to the decomposition of lignocellulosic components at higher pyrolysis temperatures (Angin et al., 2013).

**Table 2:** Proximate characterizations of cassava biochar (CBC) produced at different heating rates and temperatures.

Heating rate (°C/min)	Parameters	Pyrolysis Temperature (°C)			
		CBC-300	CBC-400	CBC-500	CBC-600
10	Moisture content (wt%)	3.46	4.25	5.40	6.00
	Volatile matter (wt%)	60.60	56.20	49.50	41.82
	Ash content (wt%)	3.50	4.30	5.0	5.80
	Fixed carbon (wt%)	32.44	35.25	40.20	46.38
20	Moisture content (wt%)	3.12	4.73	5.10	5.85
	Volatile matter (wt%)	57.80	52.60	46.20	35.10
	Ash content (wt%)	3.80	4.70	5.50	6.30
	Fixed carbon (wt%)	35.28	37.97	43.20	52.75
30	Moisture content (wt%)	2.85	3.44	4.37	5.20
	Volatile matter (wt%)	55.70	50.40	34.80	20.90
	Ash content (wt%)	4.10	5.20	5.90	7.00
	Fixed carbon (wt%)	37.35	40.96	54.93	66.90

The ash content can be described as the measure of non-volatile matter and non-combustible constituents of the biochar. As can be seen in Table 2, the ash content increased from 0.25 wt% to 3.50, 3.80 and 4.10 wt% for the heating rates of 10, 20 and 30 °C/min, respectively after the pyrolysis of the biochar at 300 °C. Further temperature increase to 600 °C at the heating rates of 10, 20 and 30 °C/min resulted in ash content increase to 5.80, 6.30 and 7.00 wt%. The increment in the ash content at higher temperatures could be linked to the steady concentration of mineral compositions (Selvarajoo et al., 2022). Typically in fuel, high ash content reduces boiler effectiveness due to enhanced ash deposition on the heat transfer surfaces, thus lower ash contents are preferable. Also, the higher quantity of ash content may enhance fouling and slagging challenges during combustion.

Furthermore, the fixed carbon content of the DCP increased from 14.77 wt% to 32.44, 35.28 and 37.35 wt% for the heating rates of 10, 20 and 30 °C/min after pyrolysis at 300 °C. Increasing the temperature up to 600 °C resulted to the higher fixed carbon content of 46.38, 52.75 and 66.90 wt% for the heating rates of 10, 20 and 30 °C/min, respectively. At elevated pyrolysis temperature, a higher number of volatile compounds are removed thereby producing a higher quantity of carbon during secondary carbonization reactions and increasing the fixed carbon content. All in all, fixed carbon is an important parameter that indicates combustion and fuel properties.

The results of the elemental analysis are presented in Table 3. According to the results, the elemental compositions of the biochar samples of CP were predominantly composed of carbon and oxygen. The carbon contents of the pyrolyzed biochar at the heating rates of 10, 20 and 30 °C/min increased from 50.44 to 52.39 wt%, 50.76 to 53.30 wt% and 50.95 to 55.44 wt% with an increase in pyrolysis temperature from 300 to 600 °C. Comparatively, hydrogen was observed to decrease at the pyrolysis temperatures of 300–600 °C from 5.62 to 5.32 wt%, 5.58 to 5.22 wt% and 5.54 to 5.05 wt% for the heating rates of 10, 20 and 30 °C/min, respectively. Also, the oxygen content of the biochar decreased from 39.76 to 35.83 wt%, 39.19 to 34.52 wt% and 38.73 to 31.87 wt% for the heating rates of 10, 20 and 30 °C/min with pyrolysis temperature increase from 300 to 600 °C.

**Table 3:** Elemental compositions of CBC produced at different heating rates and pyrolysis temperatures.

Heating rate (°C/min)	Parameters	Pyrolysis Temperature (°C)			
		CBC-300	CBC-400	CBC-500	CBC-600
10	C (wt%)	50.44	50.73	51.51	52.39
	H (wt%)	5.62	5.54	5.44	5.32
	O (wt%)	39.76	38.76	37.39	35.83
	N (wt%)	3.56	2.85	2.40	1.94
	H/C	0.11	0.11	0.11	0.10
	O/C	0.79	0.76	0.73	0.68
20	C (wt%)	50.76	51.13	51.79	53.30
	H (wt%)	5.58	5.48	5.38	5.22
	O (wt%)	39.19	38.02	36.67	34.52
	N (wt%)	3.30	2.77	2.25	1.74
	H/C	0.11	0.11	0.10	0.10
	O/C	0.77	0.74	0.71	0.65
30	C (wt%)	50.95	51.21	53.61	55.44
	H (wt%)	5.54	5.43	5.24	5.05
	O (wt%)	38.73	37.49	34.59	31.87
	N (wt%)	3.08	2.37	1.80	1.11
	H/C	0.11	0.11	0.10	0.09
	O/C	0.76	0.73	0.65	0.57

The observed decline in the hydrogen and oxygen content may be ascribed to the progressive dehydration reactions, scission of weaker bonds in biochar structure and removal of oxygenated volatile matter during biomass decomposition (Angin and Şensöz, 2014; Selvarajoo et al., 2022). In addition, the amount of nitrogen in the biochar decreased from 3.56 to 1.94 wt%, 3.30 to 1.74 wt% and 3.08 to 1.11 wt% for heating rates of 10, 20 and 30 °C/min with pyrolysis temperature increase from 300 to 600 °C. Equally, the H/C and O/C ratios were observed to decrease with temperature increase from 300 to 600 °C and may be attributed to the increasing aromaticity of the biochar. The biochars obtained at high temperatures have poor oxygen content as corroborated by the lowest O/C ratios at 600 °C and highest O/C ratios at 300 °C (Angin and Şensöz, 2014).

### 3.4 Fuel characteristics of biochar

The HHV refers to the ability of biochar to be used as fuel. According to Table 4, the HHV of the biochars increased from 22.11 to 25.00 MJ/kg, 22.56–26.14 MJ/kg and 22.87–28.70 MJ/kg for the heating rates of 10, 20 and 30 °C/min as the temperature was elevated from 300 to 600 °C.

**Table 4:** Fuel properties of cassava peels biomass (CPB) and biochar (CBC) samples.

Heating rate (°C/min)	Parameters	CPB	Pyrolysis Temperature (°C)			
			CBC-300	CBC-400	CBC-500	CBC-600
10	HHV (MJ/kg)	18.30	22.11	22.70	23.77	25.00
	Fuel Ratio	0.21	0.54	0.63	0.81	1.11
	Energy yield (%)	–	165.74	149.10	133.74	121.20
	Thermal stability	0.17	0.35	0.39	0.45	0.53
20	HHV (MJ/kg)	18.96	22.56	23.26	24.24	26.14
	Fuel Ratio	0.24	0.61	0.72	0.94	1.50
	Energy yield (%)	–	154.22	137.93	126.63	115.30
	Thermal stability	0.20	0.38	0.42	0.48	0.60
30	HHV (MJ/kg)	19.55	22.87	23.52	26.35	28.70
	Fuel Ratio	0.29	0.67	0.81	1.58	3.20
	Energy yield (%)	–	142.27	130.22	122.39	112.45
	Thermal stability	0.23	0.40	0.45	0.61	0,76

The HHV variations of the biochar samples were very small and the HHV values showed similarities with others reported from safflower seed cake, empty fruit branch and citrus peel (Angin and Şensöz, 2014; Selvarajoo et al., 2022; Selvarajoo and Oochit, 2020). The observed increment in HHV may be attributed to the increase in the biochars carbon content with increasing pyrolysis temperature, thereby leading to the intensification of mass-energy density. Overall, the HHV results in the potential application of biochar for the conversion of energy. The comparative results of HHV values for fossil fuels and biochar from different biomass materials are shown in Table 5 and the obtained results of this study indicate that CBC-600 °C biochar is highly suitable to be used as a solid fuel.

**Table 5:** HHV values comparison of solid fossil fuels and biochars.

Sample	Temperature (°C)	HHV (MJ/kg)	References
*Fossil fuels*			
Peat	–	17.00	Basu (2018a)
Lignite	–	14.00	
Bituminous coal	–	30.20	
Sub-bituminous coal	–	24.40	
Anthracite	–	32.60	
Semi-anthracite	–	29.50	
*Biochar*			
Rapeseed	700	30.47	Angin and Şensöz (2014)
Corn stover	400	23.79	Soka and Oyekola (2020a)
Cassava rhizomes	500	24.60	Rueangsan et al. (2021)
Finger millet straw	600	18.61	Karuppasamy Vikraman et al. (2021)
Castor residue	500	14.43	Kaur et al. (2018)
Mustard stalk	420	17.55	Narnaware Sunil and Panwar (2022)
Banana leaves	540	17.80	Singh et al. (2020a)
Olive stone	600	18.69	Rasam et al. (2022)
Khat stem	450	19.55	Afessa et al. (2022)
Palm fibre	500	26.60	Selvarajoo and Oochit (2020)
Coffee husk	300	25.00	Mukherjee et al. (2022)
Citrus peels	500	25.73	Selvarajoo et al. (2022)
Cassava peels	600	28.70	This study

In general, the fuel ratio is defined as the ratio of the fixed carbon to the volatile matter content. It is a significant parameter that characterizes the combustion capacity of fuel. The burning stability of the fuel is determined by the fixed carbon content, while the ignition behaviour of the fuel is determined by the volatile matter content. When the fuel ratio is high, there will be enhanced difficulty in fuel ignition that makes it burn slowly, thus causing a higher quantity of unburnt carbon that affects the combustion efficiency (Selvarajoo et al., 2022). In contrast, solid fuels with lower values of fuel ratio produce better flame stability, higher combustibility and higher carbon burnout. Therefore, the fuel ratio of the biochars was observed to increase with pyrolysis temperature as indicated in Table 2. As such, the fuel ration deserves meticulous assessment and monitoring to meet the desired value for combustion purposes.

### 3.5 Thermal stability

**Figure 3:**
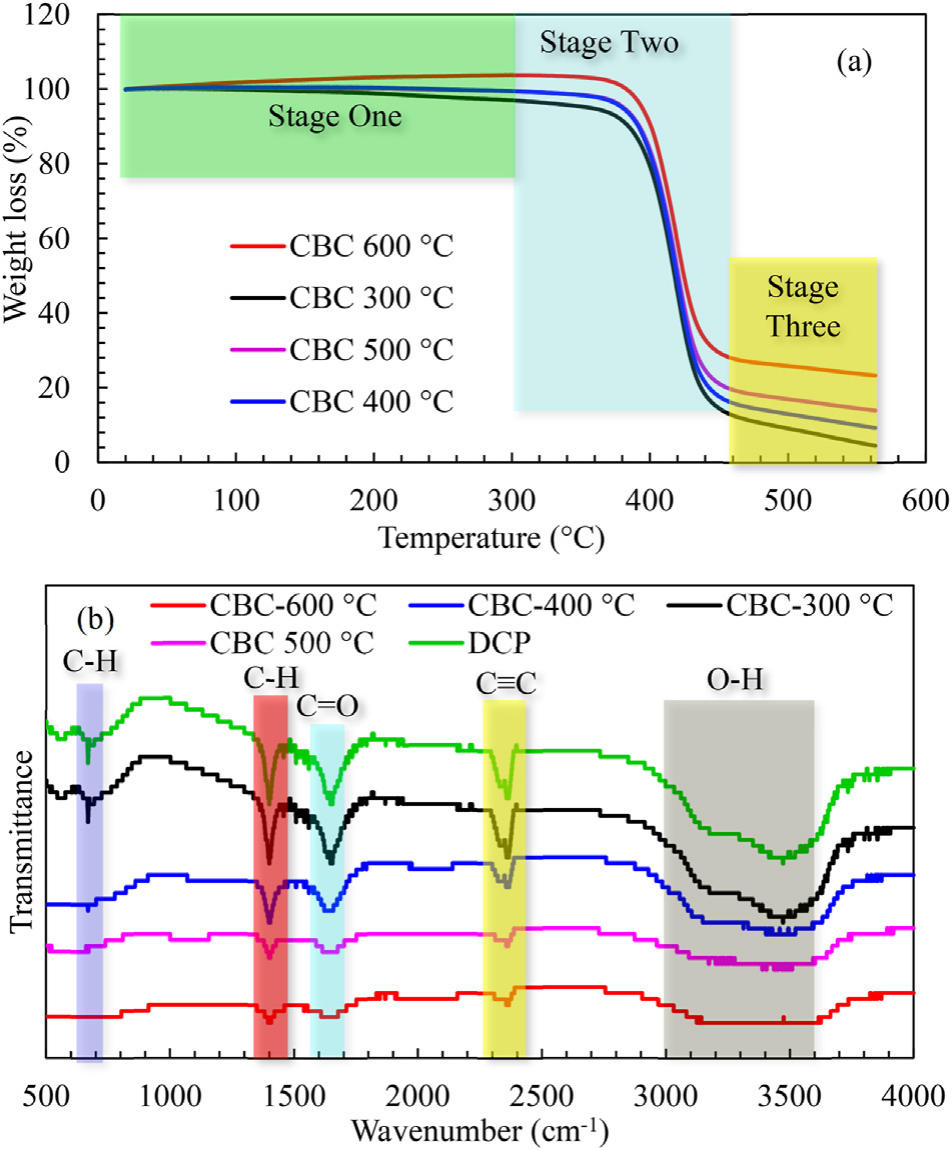
(a) TGA and (b) FTIR analysis of biochar at different temperatures and heating rate of 30 °C/min.

The TGA profiles of the pyrolyzed cassava biochar (CBC) samples are presented in Figure 3a.

The results of the TGA patterns of the CBC samples differed from that of the DCP. According to Figure 3a, three distinct stages of weight loss were observed from the thermal decomposition pattern of the CBC at different temperatures in the range of 20–300 °C, 300–460 °C and 460–580 °C, respectively. In the first stage which occurred at a temperature up to 300 °C, weight loss was ascribed to the dehydration, removal of moisture and light volatile matter from the samples (Mukherjee et al., 2022). Particularly, the observed weight loss associated with dehydration was not significant in all the samples and may be attributed to the hydrophobic nature of the biochars compared to the DCP sample. Notably, rapid weight loss was observed in the second stage at the temperature range of 300–460 °C for CBC-300 °C, CBC-400 °C, CBC-500 °C and CBC-600 °C, respectively. The significant mass loss observed at this temperature zone may be attributed to the combustion of volatile matters and is considered the active phase of the pyrolysis process (Askeland et al., 2019). Generally, the basic lignocellulosic building blocks decomposition is obtained for hemicellulose (220–315 °C), cellulose (325–400 °C) and lignin (400–900 °C) (Mukherjee et al., 2022). The decomposition of hemicellulose occurred in stage one, while hemicellulose, cellulose and lignin decomposed in stage two. Also, the decomposition of only lignin continued in stage three as shown in Figure 3a. The maximum weight loss recorded in the second stage are 87%, 81%, 75% and 65% for CBC-300 °C, CBC-400 °C, CBC-500 °C and CBC-600 °C, respectively.

The mass loss observed in the third stage of the decomposition pattern (460–580 °C) was linked to char combustion and oxidation (Selvarajoo et al., 2022). In the third decomposition pattern, the lowest reactivity and combustion of residue fixed carbon was observed with a decline in the rate of weight loss. The observed decline in mass loss trend may be linked to the attainment of thermal stability by the CBC samples at different temperatures. Generally, the thermal stability of biomass materials is achieved under severe pyrolysis conditions of higher pyrolysis temperature, higher heating rate and longer residence time (Mukherjee et al., 2022). Above all, higher mass loss was recorded in stage two for biochar at a lower pyrolysis temperature and may be attributed to the presence of a large number of combustible materials in the biochar that was possibly left behind due to incomplete decomposition of lignocellulosic materials during pyrolysis (Selvarajoo et al., 2022). In this study, CBC-600 was obtained as the most thermally stable at the heating rate of 30 °C/min compared to other biochar samples. The results of this study are consistent with the literature that reported higher thermal stability when combusted for biochar produced at higher pyrolysis temperature (Askeland et al., 2019; Tomczyk et al., 2020; Zhao et al., 2017). In biochar, higher thermal stability is denoted as the capacity to withstand higher combustion temperature for a particular time without indicating a significant decomposition (Selvarajoo et al., 2022). Typically, biochar indicates a better combustion behaviour due to the presence of active sites in the porous surface of biochar that is easily assessed by the reactive gas, unlike the biomass. Fixed carbon combustion represents the predominant biochar combustion, while the dominant combustion process for biomass is ascribed to volatilization and gas phase combustion due to high volatile matter and fixed carbon ratio (Selvarajoo et al., 2022).

### 3.6 Functional groups analysis

To evaluate the influence of pyrolysis conditions on the chemical structure of DCP and CBC samples, FTIR analysis was carried out as shown in Figure 3b. The identified sharp peak at 670 cm-1 was ascribed to the occurrence of C–H out of plane bend for aromatic compounds. The observed peak at this region was pronounced in DCP and CBC-300 °C samples but narrowed in CBC-400 °C, CBC-500 °C and disappeared in the CBC-600 °C pyrolyzed sample. Transmittance peaks observed at 1400 and 1650 cm^−1^ in both DCP and CBC samples pyrolyzed at different temperatures correspond to the asymmetric C–H bend and C=O stretch due to the presence of methylene group (-CH_2_) of the alkane compounds and carbonyl group of the aldehyde or ester compounds (Selvarajoo et al., 2022). Importantly too, **t**he vibrational intensity of the peaks was observed to decrease as the pyrolysis temperature increased. This trend reveals the progressive decomposition of the alkane and carbonyl groups in the biomass and biochar samples at different temperatures, thereby demonstrating a chemical change from the cellulose and hemicellulose decomposition (Mukherjee et al., 2022). The peak at 2357 cm^−1^ was prominent on the DCP and CBC-300 °C samples, but significantly decreased in CBC-400 °C, CBC-500 °C and CBC-600 °C pyrolyzed biochar samples. The detected peak corresponds to the C≡C stretch of the saturated aliphatic (alkane) compounds. Similarly, the transmittance peaks at this region may also suggest the presence of transition metals of carboxylic, acetylenic and carbonyls compounds.

The broad peaks detected at 3200 and 3490 cm^−1^ corresponded to the O–H stretching of the hydroxylic, carboxylic and alcoholic groups. In this transmittance region, the peaks decreased with increasing pyrolysis temperature which may be attributed to reactions due to dehydration (Selvarajoo and Oochit, 2020). Above all, FTIR spectra of the DCP and CBC samples revealed the heterogeneous nature of the samples by the detection of several functional groups. In this study, the most significant peaks among all the biochar samples were indicated by CBC-300, demonstrating the stronger presence of the functional groups, while CBC-600 showed the weakest peaks presence. Overall, the effects of pyrolysis temperature on FTIR profile were pronounced due to the observed reduction in the spectra vibrational intensity with increasing pyrolysis temperature. This indicates that increasing the pyrolysis temperature resulted in a decrease in H–C and O–C bonds, thereby enhancing the hydrophobicity of biochar which may be ascribed to the dehydration and deoxygenation reactions (Mukherjee et al., 2022). Generally, fuel with higher hydrophobicity is preferable due to its improved moisture resistance. Therefore, a low humidity content is recommended to achieve a higher combustion efficiency of biochar fuels.

### 3.7 Surface morphology

**Figure 4:**
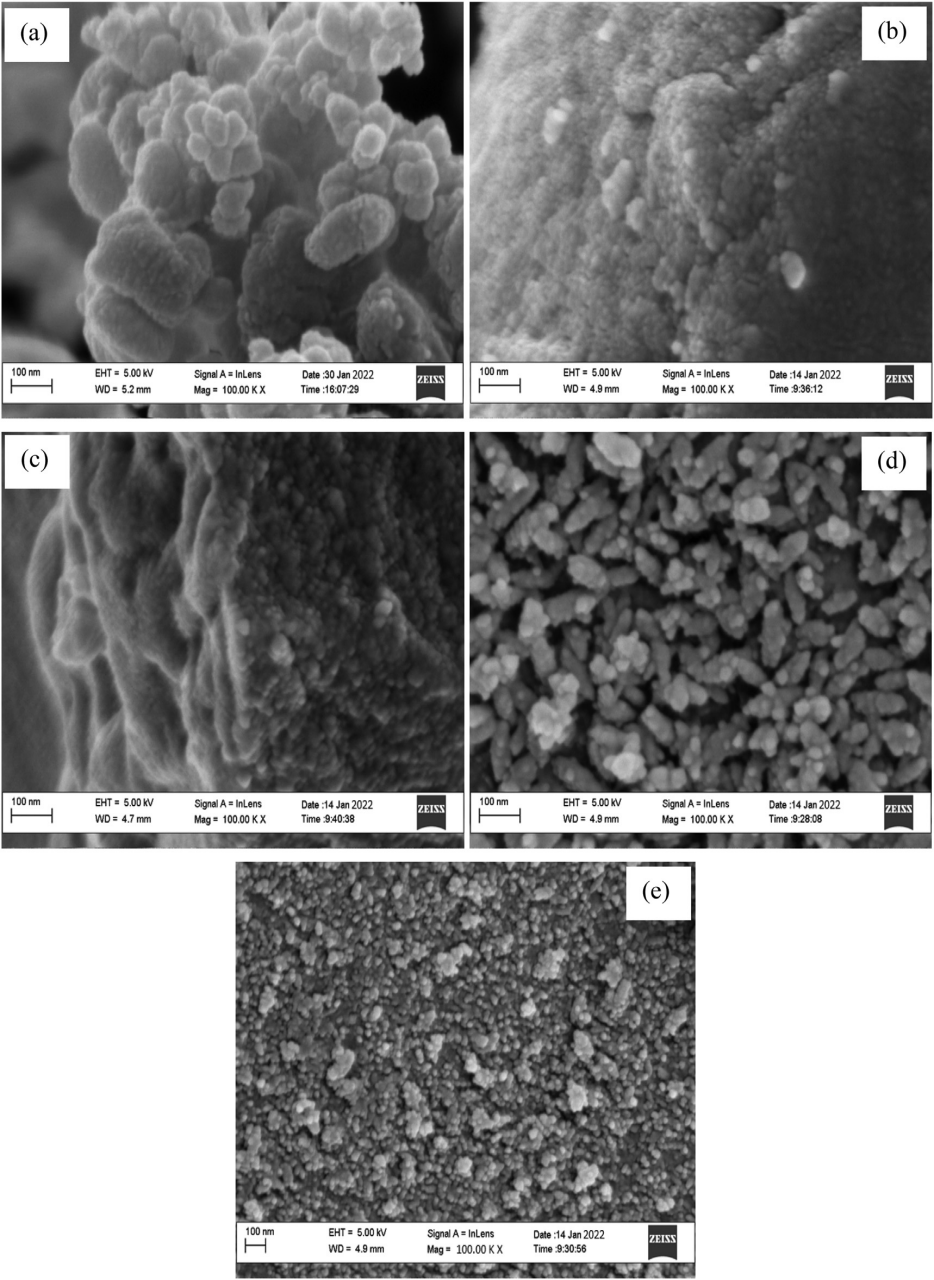
Images of HRSEM for (a) DCP; biochar at (b) 300 °C; (c) 400 °C; (d) 500 °C and (e) 600 °C (heating rate = 30 °C/min).

The investigation of surface morphology of DCP and NiNPs catalyzed CBC at different pyrolysis temperatures was investigated using high-resolution scanning electron microscopy (HRSEM) and the results are shown in Figure 4(a-e). As can be seen in Figure 4a, the surface structure of DCP was visibly rough and slightly porous but was not defined properly as the biochars (CBC samples). This may be due to the small quantity of volatile matter that was lost through the drying process during the preparation stage of the samples. According to Figure 4b, the porous surface of CBC-300 °C was not well defined in comparison with the biochars produced at higher temperatures (CBC-400 °C, CBC-400 °C and CBC-600 °C). Hence, the structural changes on the NiNPs catalyzed CBC were very evident at higher pyrolysis temperatures. The occurrence of few pores on the surface of CBC-300 °C may be attributable to the inability of the organic components to completely decompose due to the poor diffusion of NiNPs catalysts at low temperatures (Egbosiuba et al., 2020b).

Overall, the NiNPs assisted degradation process enabled the formation of characteristic openings, pores and voids on the surface of biochars through the release of volatile matter and catalytic decomposition of the lignocellulosic material (Egbosiuba and Abdulkareem, 2021). Further increase in the pyrolysis temperature as shown in Figure 4(c,d,e) revealed increasing development of pores and enhancement of the existing well-defined pores through cell-wall distortions and decomposition of solid surface by the NiNPs catalyzed process. The observed increase in the amount of pore formation with temperature increase significantly improves the surface area of biochars, whereby biochars with a high surface area are highly desired for soil enrichment and carbon sequestration.

Furthermore, pore sizes on the surfaces of CBC-300 °C, CBC-400 °C, CBC-500 °C and CBC-600 °C decreased with an increase in the pyrolysis temperature and the reduction in the pore sizes may be ascribed to the formation of more micropores at higher pyrolysis temperature. In this study, it can be concluded that NiNPs assisted pyrolysis of cassava peels at elevated temperature favours the formation of a large number of micropores, indicating an improvement in the surface area of the biochar samples. Similarly, structural change in biomass morphology and the degradation of the biochar structure under the catalytic influence at higher temperatures, higher heating rates and longer residence time had been reported in the literature (Askeland et al., 2019; Mukherjee et al., 2022; Nkomo et al., 2021).

### 3.8 Fuel characteristics of bio-oil

The physical and chemical characteristics that define the fuel properties of bio-oil such as kinematic viscosity, density, pH, HHV and flash point were determined and the result is presented in Table 6.

**Table 6:** Properties comparison of bio-oil and conventional diesel.

Heating rates	Temperature	Properties				
		Kinematic viscosity @25 °C (mm^2^/s)	Density (g/cm^3^)	pH	HHV (MJ/kg)	Flash point (min)
		Conventional diesel values (ASTM D975, 2013)				
		2–4	0.815–0.870	2–5	30–43	52–60
		This study				
10	300	3.30	0.823	2.35	38.24	58.20
	400	2.98	0.820	2.68	38.57	57.36
	500	2.45	0.819	3.10	39.10	57.14
	600	2.10	0.816	3.77	39.55	56.38
20	300	3.53	0.833	2.98	38.42	56.31
	400	3.31	0.828	3.31	38.90	56.15
	500	2.96	0.825	3.90	39.65	55.73
	600	2.32	0.818	4.50	40.80	55.10
30	300	3.87	0.850	3.22	38.10	55.41
	400	3.55	0.841	3.85	40.55	54.50
	500	3.12	0.829	4.30	41.12	54.22
	600	2.90	0.832	4.96	42.68	53.85

In this study, the kinematic viscosity of bio-oil was tested using a kinematic viscosity bath (BABIR-KVB001, India) at 25 °C. The viscosity of the bio-oil was in the range of 2.10–3.87 mm^2^/s (Table 6) and showed correspondence with the conventional diesel fuels standard. The observed viscosity range of the bio-oil indicates the possibility of minimized problems of clogging and coking at the cooling device. Due to the presence of water in bio-oil, it undergoes polymerization reactions, whereby the speed of reaction increases with temperature increase and the viscosity also reduces with temperature increase (Li et al., 2021).

Herein, the fuel properties of the bio-oil were also determined. For instance, the density of bio-oil is a very significant parameter for transportation, storage and bio-oil usage. From the result of the bio-oil density presented in Table 6, the density of bio-oil decreased with increasing temperature and heating rates. The observed reduction in density at higher pyrolysis temperature and heating rates may be attributable to the loss of water molecules, thereby making it more convenient to use and transport which invariably reduces cost and increases efficiency (Li et al., 2021).

In the storage of bio-oil, propylene and acid-resistant stainless steel are mostly used. Bio-oil may cause damage to the internal combustion engine when used as a liquid fuel without treatment due to its corrosive nature. To obtain the pH of bio-oil, a Hanna pH meter was used and the results in Table 4 revealed a pH range of 2.35–4.96 at the various temperatures and heating rates. Importantly too, the obtained pH in this study falls within the prescribed conventional fuel standards by ASTM and in the literature. The observed acidic pH of the bio-oil may be ascribed to the presence of small molecular organic acids such as pyruvic acid, propionic acid, and acetic acid.

The HHV is an important fuel property of bio-oil because it takes a lot of heat to ignite due to the presence of non-volatile components. The HHV of the bio-oil shown in Table 4 indicated that the value increased with increasing temperatures and heating rates from 38.24 to 42.68 MJ/kg. The obtained results fall within the conventional fuel range and are very suitable for being used as fuel oil and in boiler combustion. The HHV values compared significantly with the literature and the low HHV at low temperatures may be due to the presence of water that causes ignition problems. Further dehydration and deoxidization may be required to enhance the widespread application of bio-oil. In addition, the flash point of the bio-oil was evaluated using Cleveland open cup method and the result is shown in Table 6. Accordingly, the flash point revealed a decreasing trend with increasing temperature at the various heating rates. Above all, the fuel properties of the bio-oil showed remarkable properties that indicate its suitability as an alternative to diesel fuel.

### 3.9 The catalytic mechanism of the pyrolysis process

**Figure 5:**
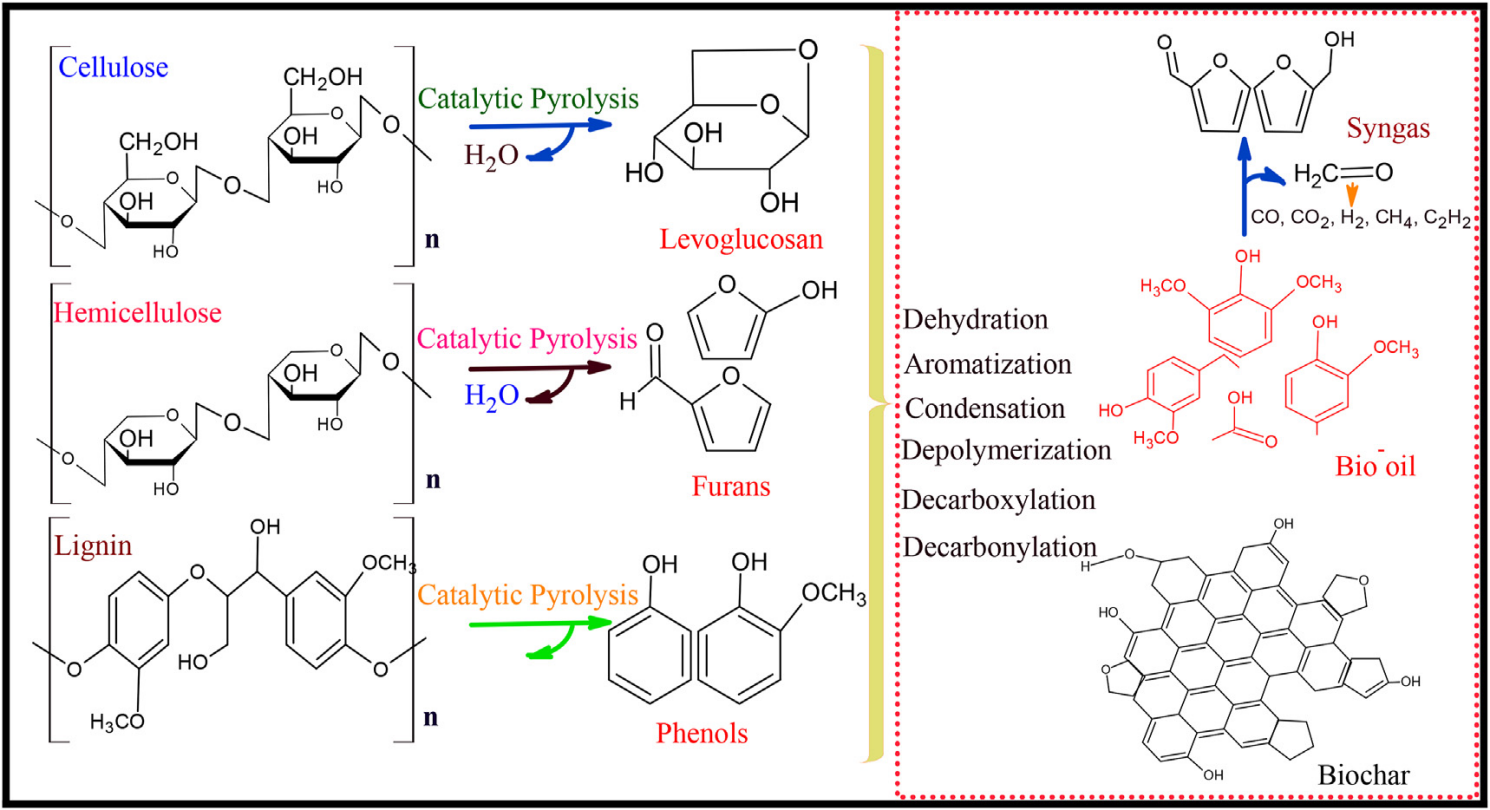
The formation mechanism of the catalytic pyrolysis.

The NiNPs catalyzed pyrolysis reaction mechanism is presented in Figure 5. The CBC showed highly porous biochar due to the effect of NiNPs catalyst at a higher temperature. During the catalyzed pyrolysis, the improved production of gaseous species such as CO, CO_2_ and H_2_ enabled structural modifications of the biochar which subsequently evaporates to enhance additional pores that are primarily microporous (Luo et al., 2022b; Zhang et al., 2022b).

The degradation of the cellulose, hemicellulose and lignin under the catalyst influence enhanced the microporosity of the biochar as also corroborated by the morphological structures of the CBC in Figure 4. In the pyrolysis process, NiNPs reduced the activation energy of the reaction due to its transition origin and promoted the fracture of C–C and C–H bonds (Luo et al., 2022a). The cracking of primary volatiles into various short chain alkenes is also facilitated by a large number of free radicals, thereby generating smaller molecules due to the strong acid sites between the metal and biochar (Ghodake et al., 2021). The pyrolysis mechanism indicates the formation of aromatic hydrocarbons, liquid oil compounds and alcohols through dehydration, polymerization, aromatization and decarboxylation. The formation of biochar may be ascribed to the reaction of CO disproportionation and CH_4_ dehydrogenation. Further cracking is also facilitated by the catalyst through decarboxylation and decarboxylation to enhance the contents of H_2_ and CO_2_. In the end, the NiNPs catalysts significantly promoted the conversion of DCP into biochar and bio-oil as a dependable quality fuel source. The catalyzed pyrolysis mechanism can provide a reliable guide in catalyst selection and the realization of the desired product distribution.

## 4 Conclusions, limitations and future prospects

### 4.1 Conclusion

Thermogravimetric analysis was successfully utilized to obtain the thermal decomposition temperature range of dried cassava peels at different heating rates. Dried cassava peels were successfully pyrolyzed over a temperature range of 300–600 °C and the heating rates of 10, 20 and 30 °C/min through slow pyrolysis to produce solid fuel biochar and liquid fuel bio-oil as the main product. The product distribution showed a decrease in the biochar yield with increasing temperature and heating rates. However, the yield of bio-oil increased with temperature and heating rates. The volatile matter content decreased with temperature increase at different heating rates. The HHV of the biochars increased for the different heating rates at the elevated temperature of 600 °C. The FTIR confirmed the decreasing trend of spectra with increasing temperature. The TGA and HRSEM analysis revealed that biochar produced at 600 °C showed better thermal stability. Furthermore, the fuel properties of bio-oil such as kinematic viscosity, density, pH, HHV and flash point were of good quality compared to conventional diesel standards. Cassava peels have shown a remarkable potential to be reused and upgraded to biochar and bio-oil to achieve energy conversion.

### 4.2 Limitations and future work

Many nations around the globe are keying into the carbon reduction policy to achieve a carbon-neutral society enhanced by green technology. For this purpose, this study projects the importance of human development in the management of agricultural wastes to achieve a safe and healthy environment. However, the challenges of this research include the application of high-value pyrolysis products, reuse of catalysts and coke deactivation, the possibility of new problems emanating in the commercialization of the catalyzed pyrolysis process and lack of economic costs and environmental implications considerations. Future work may concentrate on the application of the pyrolysis products, deactivation of the catalysts, catalysts regeneration and experimental scale-up to industrial and commercial stages. In this study, the quality of biochar and bio-oil were significantly improved, but the estimation of gaseous products for effective understanding of the reactions that occurred during the catalyzed pyrolysis process requires additional research. Also, further evaluation of the combination of exergy-based techniques and sustainability tools (such as life cycle assessment (LCA) and thermodynamics shared energy) may present a promising method for concurrent analysis of biofuel production from economic, environmental and thermodynamic viewpoints.

## Declarations

### Author contribution statement

Titus Chinedu Egbosiuba: Conceived and designed the experiments; Performed the experiments; Analyzed and interpreted the data; Contributed reagents, materials, analysis tools or data; Wrote the paper.

### Funding statement

This research did not receive any specific grant from funding agencies in the public, commercial, or not-for-profit sectors.

### Data availability statement

Data will be made available on request.

### Declaration of interests statement

The authors declare no conflict of interest.

### Additional information

No additional information is available for this paper.
